# Evaluation of the trūRapid^™^ FOUR heartworm antigen test: a reliable point-of-care diagnostic tool for heartworm detection in dogs

**DOI:** 10.1186/s13071-026-07630-8

**Published:** 2026-08-27

**Authors:** Haichen Song, Chinta M. Lamichhane, Xiuli Yang, Meera Surendran-Nair, Frances Moore, Graham Bilbrough, Michelle D. Evason, Demitria M. Vasilatis, Jennifer L. Willcox, Christian M. Leutenegger, Pablo D. Jimenez Castro

**Affiliations:** Mars Petcare Science & Diagnostics, Antech Diagnostics, Loveland, CO USA

**Keywords:** *Dirofilaria immitis*, Heartworm antigen, trūRapid^™^ FOUR, DiroCHEK^®^, SNAP^®^ 4DX^®^ Plus, Lateral flow immunoassay, Point-of-care diagnostics

## Abstract

**Background:**

Heartworm disease, caused by Dirofilaria immitis, is a globally distributed and clinically significant cardiopulmonary condition affecting dogs and other animal species. Dogs typically lack overt clinical signs in the early stages of infection, and disease progression can lead to worsening clinical signs and poor prognosis. The purpose of this study was to evaluate the performance of the trūRapid™ FOUR heartworm antigen test (Antech Diagnostics) for the detection of D. immitis antigen in canine serum samples and to compare its performance with another commercially available point of care (POC) heartworm antigen test, specifically the SNAP® 4DX® Plus (IDEXX), using DiroCHEK® (Zoetis) as the reference test.

**Methods:**

A total of 350 canine remnant serum samples were evaluated using trūRapid™ FOUR, SNAP® 4DX® Plus, and DiroCHEK® as per manufacturers' instructions.

**Results:**

For samples positive at DiroCHEK® (n = 149), the sensitivity of the trūRapid™ FOUR heartworm antigen test was 98.7% (95% CI 94.3-99.6%), with a specificity of 100% (95% CI 98.2-100%) for samples negative at DiroCHEK® (n = 201). The trūRapid™ FOUR showed excellent agreement with another available POC test, the SNAP® 4DX® Plus (0.994 ? agreement, 95% CI 0.983-1.000).

**Conclusions:**

The findings demonstrate that the trūRapid™ FOUR heartworm antigen test provides reliable diagnostic accuracy, ease of use, and a fast time to result (10 min), making it a robust POC option for detecting canine heartworm infection. Its performance is comparable to the reference laboratory ELISA test, DiroCHEK®, and another commercially available POC test, SNAP® 4DX® Plus. Overall, the results support the use of the trūRapid™ FOUR heartworm antigen test as a dependable clinical tool for the detection and management of heartworm infection in dogs.

**Graphical Abstract:**

**Figure:**
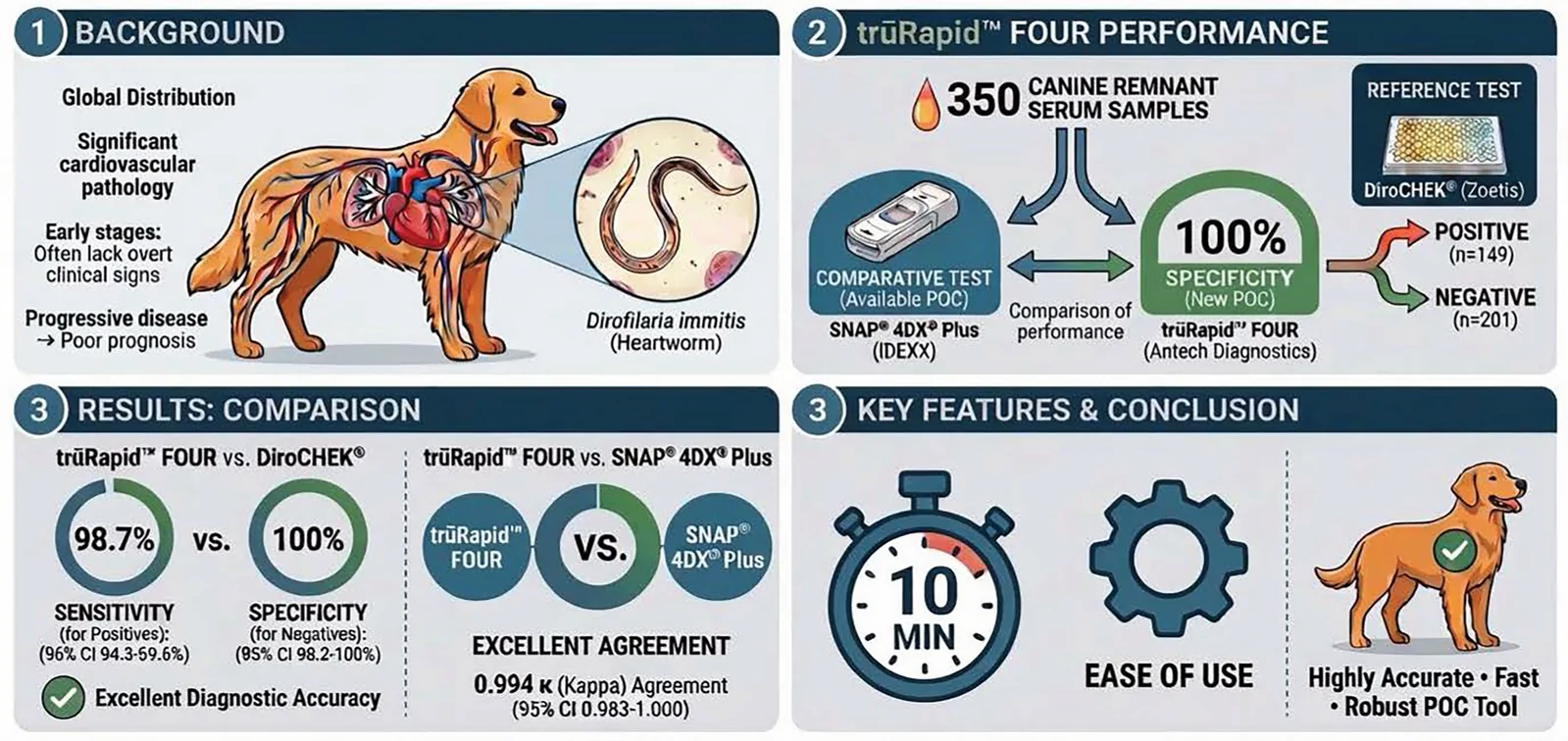

## Background

Heartworm disease (*Dirofilaria immitis*) is one of the most clinically significant vector-borne disease concerns affecting dogs globally [1]. Infection rates range from sporadic cases in non-endemic areas to high prevalence in tropical, subtropical, and temperate regions, where environmental conditions favor sustained transmission [2–5]. The national average incidence of heartworm infections in dogs in the United States is 0.94%, with tropical regions reporting even higher incidence of infections [6, 7]. In dogs, the parasite is transmitted through mosquito vectors belonging to *Culex*, *Aedes*, and *Anopheles* genera, and infection causes progressive damage to the pulmonary arteries and right side of the heart [5]. The life cycle of *Dirofilaria immitis* is relatively long (usually 7–9 months) compared with most parasitic nematodes [8]. Other hosts, such as cats and ferrets, are less suitable, and they may develop single-sex infections or low-level, short-lived microfilaremia and play only a limited role in transmission [1]. Infections in humans have also been described in different organ systems and in different parts of the world [9].

Given the global scope of heartworm infection, diagnostic and screening recommendations should be interpreted in a regional context; in addition to North American guidance from the American Heartworm Society (AHS) and Companion Animal Parasite Council (CAPC), across Federation of Companion Animal Veterinary Associations (FECAVA) and European Scientific Counsel Companion Animal Parasites (ESCCAP)-endorsed European recommendations and the Australian Heartworm Advisory Panel (AHAP), antigen testing is universally regarded as the primary screening method, ideally complemented by microfilaria detection to improve diagnostic sensitivity and provide epidemiological insight [10–14]. Overall, the guidelines highlight a shared global consensus on the importance of combined diagnostic approaches, pre-treatment screening, and context-specific testing strategies.

Medical and surgical management of canine heartworm disease can be difficult, and some animals experience long-term sequelae from infection-related damage [15–17]. Despite the possibility of severe clinical signs, dogs typically present with no clinical signs, underscoring the importance of regular screening [10, 11].

Diagnostic approaches for *D. immitis* infection include direct detection of microfilariae by modified Knott’s test, antigen detection via enzyme-linked immunosorbent assay (ELISA), and rapid point-of-care (POC) lateral-flow immunoassays [10, 11]. Heartworm antigen is generally detectable around 5 months after infection, while microfilariae usually appear by 6 months. Antigen tests are developed to recognize the antigen of adult female heartworms even in amicrofilaremic dogs. Previously reported sensitivity and specificity were high for antigen tests broadly, with as high as 100% sensitivity and 100% specificity described, depending on test type and worm burden [18–22]. Positive results are then followed by confirmatory tests with high specificity, which help rule out false positives and establish a definitive diagnosis [23]. In contrast, when diagnosing disease in clinically suspected cases (rule-in testing), where the pathogen burden is typically high, tests with high specificity are especially valuable, because they provide greater confidence that a positive result truly indicates disease [20–23]. Due to their high diagnostic accuracy, antigen tests are widely used by veterinarians both for routine heartworm screening in dogs with no clinical signs and for rule-in heartworm infections in clinically suspected patients. However, a negative result (no antigen detected) does not definitively rule out heartworm infection. A false negative result may occur in dogs with low worm burdens, sample collection during the pre-patent period (larvae or immature adults), all-male infections, or when antigen is bound within immune complexes [18, 19, 22, 24–26].

DiroCHEK^®^ is a plate-based reference assay widely used in diagnostic laboratories to confirm *D. immitis* infection. Although it is highly specific and demonstrates 100% sensitivity in dogs harboring three or more adult female heartworms, its diagnostic accuracy can be affected by low worm burden, immature female, or male-only infections, similar to other POC antigen tests [18, 19, 26]. DiroCHEK^®^ assay format requires the use of serum or plasma samples and must be performed by trained technicians using laboratory equipment, while POC heartworm antigen tests, such as SNAP^®^ 4Dx^®^ Plus and trūRapid^™^ FOUR, can detect *D. immitis* in anticoagulated whole blood, serum, and plasma, and require no other consumables to run [27–29]. This wider sample range and ease-of-use format provide veterinary professionals with vital tools in diagnosing and monitoring canine heartworm infection and disease. Although all three diagnostic tests can be read visually or with an optional reader, they differ in format, detection method, and storage temperature requirements. Both DiroCHEK^®^ and SNAP^®^ 4Dx^®^ Plus are ELISA-based assays requiring refrigerated storage at 4–8 °C [27, 28, 30]. In contrast, trūRapid™ FOUR is a lateral-flow test that can be stored at room temperature [31]. The purpose of the study is to demonstrate that the POC test, such as trūRapid^™^ FOUR, is comparable to the more resource- and time-intensive test available at a reference laboratory for rule-in testing against *D. immitis* infection.

## Methods

### Study design

The diagnostic accuracy study was conducted using a retrospective panel of canine remnant serum samples and represents a single-gate cross-sectional study design [32]. All samples had been previously submitted to a U.S. veterinary diagnostic laboratory from heartworm-endemic regions of the southeastern United States. The study was designed to evaluate the performance of the trūRapid^™^ FOUR test compared with the SNAP^®^ 4DX^®^ Plus test, using the DiroCHEK^®^ as the reference standard.

### Samples

A total of 350 canine serum remnant samples were collected from heartworm-endemic regions of the southeastern United States, including Florida and North Carolina. All samples were previously tested using DiroCHEK^®^. The samples were collected over time in the past decade and selected based on prior test results and available volume to ensure that both heartworm-positive and heartworm-negative samples were adequately represented in the sample panel. Samples were stored at −80 °C until testing. Sample status was blinded to the tester performing the test.

### Comparative testing of the heartworm antigen tests

Each sample was tested as a single replicate with trūRapid^™^ FOUR (Antech Diagnostics, Mars Petcare Science & Diagnostics, Loveland, CO, USA), SNAP^®^ 4Dx^®^ Plus (IDEXX, Westbrook, ME, USA), and DiroCHEK^®^ (Zoetis, Parsippany, NJ, USA) according to the manufacturer’s instructions. Results for trūRapid^™^ FOUR test and SNAP^®^ 4Dx^®^ Plus test were read at 10 min and 8 min, respectively, following sample application. The test results were based on visual interpretation. Any visible test band/spot, regardless of intensity, was considered antigen-positive, whereas the absence of a visible test band/spot in the reading window was interpreted as no detectable antigen. Tests with no control band/spot were recorded as invalid test results, and no repeated test was performed. The invalid test results were reported in the final analysis. For the DiroCHEK^®^ reference standard, only visual interpretation was obtained; the sample was considered antigen-positive if a clear to blue color change occurred within 5 min of adding the final solution. Otherwise, the sample was considered to have no detectable antigens for heartworm infection. Results were compared among these three tests using DiroCHEK^®^ as the reference standard.

### Statistical analysis

Diagnostic accuracy metrics were calculated using DiroCHEK^®^ results as the reference standard. Sensitivity and specificity of the trūRapid^™^ FOUR test were computed with corresponding 95% confidence intervals (CIs). Agreement between the trūRapid^™^ FOUR and SNAP^®^ 4DX^®^ Plus tests was assessed using Cohen’s kappa (*κ*) statistic with 95% CIs and interpreted according to standard criteria: almost perfect agreement for *κ* = 0.81–1.00, substantial agreement for *κ* = 0.61–0.80, and moderate agreement for *κ* = 0.41–0.60 [33]. All analyses were performed using standard statistical methods for evaluating diagnostic accuracy.

Sensitivity was calculated as TP/(TP + FN) and specificity as TN/(TN + FP), where TP = true positives, FN = false negatives, TN = true negatives, and FP = false positives. Agreement beyond chance was assessed using Cohen's kappa (*κ*). *κ* was calculated as (*P*_o_−*P*_e_)/(1−*P*_e_), where *P*_o_ is the observed proportion of agreement, and *P*_e_ is the proportion of agreement expected by chance [34]. Ninety-five percent confidence intervals for sensitivity and specificity were calculated using the Wilson method [35], and 95% confidence intervals for Cohen's kappa were calculated using the binomial method [36].

## Results and discussion

All assays were completed successfully, and no invalid, indeterminate, or missing results occurred for any of the three tests. Therefore, all 350 samples were included in the final analysis. Of 350 canine serum samples, 149 were positive, and 201 were negative for heartworm antigen by DiroCHEK^®^. The trūRapid^™^ FOUR heartworm antigen test correctly identified 147 of 149 positives and all 201 negatives, yielding a sensitivity of 98.7% (95% CI 94.3–99.6) and specificity of 100% (95% CI 98.2–100.0). The SNAP^®^ 4Dx^®^ Plus identified 148 of 149 positives and all 201 negatives, demonstrating a sensitivity of 99.3% (95% CI 96.3–100.0) and a specificity of 100% (95% CI 98.2–100.0) compared to DiroCHEK^®^ (Table 1). Direct comparison between trūRapid™ FOUR and SNAP^®^ 4Dx^®^ Plus showed near-perfect agreement, with a Cohen’s kappa coefficient of 0.994 (95% CI 0.983–1.000) (Table 1).

**Table 1:** Comparative performance of trūRapid^™^ FOUR and SNAP^®^ 4Dx^®^ Plus heartworm antigen tests in dogs using DiroCHEK^®^ as reference test

Test comparison	Positive (*n* = 149)	Negative (*n* = 201)	Sensitivity (%) (95% CI)	Specificity (%) (95% CI)	*κ* (95% CI)
trūRapid^™^ FOUR vs DiroCHEK^®^	147	201	98.7 (94.3–99.6)	100 (98.2–100.0)	0.988 (0.972–1.000)
SNAP^®^ 4Dx^®^ Plus vs DiroCHEK^®^	148	201	99.3 (96.3–100.0)	100 (98.2–100.0)	0.994 (0.983–1.000)
trūRapid™ FOUR vs SNAP^®^ 4Dx^®^ Plus	NA	NA	NA	NA	0.994 (0.983–1.000)

Sensitivity and specificity were calculated using DiroCHEK^®^ as the reference standard (*n* = 149 positive; *n* = 201 negative). *NA* not applicable, *κ* Cohen’s kappa coefficient, *CI* confidence interval

The results of this study indicate that the trūRapid^™^ FOUR heartworm antigen test achieves a diagnostic performance comparable to the reference DiroCHEK^®^ ELISA and the commercial POC test, SNAP^®^4Dx^®^Plus. The sensitivity (98.7%) and specificity (100%) reported are consistent with previous reports of high-quality antigen assays [18–22]. High test specificity prevents unnecessary confirmatory testing in false-positive cases, while sensitivity close to 100% ensures accurate detection of infected dogs even in the absence of microfilaria. The findings of the study align with recent clinical studies showing that the trūRapid^™^ assay exhibits diagnostic performance comparable to that of established point-of-care tests, including SNAP^®^ 4DX^®^ Plus, in the evaluation of suspected heartworm infections [21].

Routine screening for canine heartworm infection is a cornerstone of the AHS prevention strategy, with annual antigen testing recommended for all dogs receiving heartworm preventives, irrespective of perceived compliance or previous negative test results [10]. However, this emphasis on routine annual screening is not uniformly reflected in international guidelines. While CAPC, ESCCAP, and AHAP all recommend antigen testing as the primary diagnostic method, ideally complemented by microfilaria detection, their recommendations generally adopt a more risk-based approach in which testing frequency is guided by local heartworm prevalence, vector distribution, travel history, and individual patient risk [11–14]. These regional differences primarily reflect variation in disease endemicity, mosquito ecology, climatic conditions, preventive practices, and healthcare resource allocation rather than disagreement regarding diagnostic principles. Consequently, although there is broad international consensus on the use of combined antigen and microfilaria testing for the diagnosis of canine heartworm infection, recommendations for routine screening frequency should be interpreted within the context of regional epidemiology and local prevention strategies [10–14].

DiroCHEK^®^ is recognized for its excellent analytical sensitivity in established heartworm infection but requires laboratory equipment and trained technicians. Commercial POC tests such as SNAP^®^ 4Dx^®^ Plus and trūRapid^™^ FOUR extend diagnostic capability to the clinical setting by providing rapid, instrument-free results within minutes. In addition, both POC tests allow simultaneous detection of antibodies to three other major vector-borne pathogens in a single sample (*Borrelia burgdorferi*, *Ehrlichia* spp. and *Anaplasma* spp.), allowing comprehensive vector-borne disease pathogen screening [28, 29]. While SNAP^®^ 4Dx^®^ Plus utilizes a flow-through ELISA platform, trūRapid™ FOUR employs lateral-flow technology [29]. The assay produces results in approximately 10 min, requires no instrumentation, and is stable at ambient temperature for its entire shelf life—key features for busy veterinary clinics and mobile practices [31]. The resulting near-perfect κ agreement (*κ* = 0.988) reflects excellent clinical equivalence to DiroCHEK^®^.

This study was limited by the use of archived canine serum samples, for which information on the true infection status, adult worm burden, and stage of infection was unavailable. In addition, the diagnostic sensitivity of heartworm POC tests may be underestimated when archived sera are used [37]. Consequently, the diagnostic performance of the trūRapid^™^ FOUR test could only be evaluated against the DiroCHEK^®^ reference assay rather than a definitive determination of infection status. Although DiroCHEK^®^ is a well-established laboratory assay, its diagnostic performance may be compromised in dogs with low worm burdens, infections with immature female worms, or male-only infections. Therefore, it cannot be considered a definitive indicator for true infection status.

Although the adult worm burden was unknown for the 149 DiroCHEK^®^ positive samples included in this study, the samples were expected to represent a range of worm burdens encountered in routine diagnostic submissions. Furthermore, trūRapid^™^ FOUR has recently been shown to have sensitivity comparable to that of SNAP^®^ 4Dx^®^ Plus and DiroCHEK^®^ for the early detection of heartworm antigen in experimentally infected dogs [38], supporting the comparable diagnostic performance observed in the present study. Future prospective studies involving dogs with confirmed infection status, quantified adult worm burdens, known heartworm preventive histories, and microfilaremia status are warranted to further evaluate the clinical performance and utility of the trūRapid^™^ FOUR test.

## Conclusions

The results demonstrate that the performance of the trūRapid^™^ FOUR heartworm antigen test is comparable to the reference laboratory ELISA test and another commercially available POC test for rule-in infection. The test provides comparable diagnostic sensitivity and specificity to DiroCHEK^®^, with ease of use and a fast time-to-result (10 min), making it a reliable POC option for veterinary clinics and mobile practices.

## Data Availability

Data supporting the main conclusions of this study are included in the manuscript.
